# Supplementary material for: Patterns of Intron Gain and Loss in Fungi
Source: PLoS Biol. 2004 Nov 30;2(12):e422. doi: 10.1371/journal.pbio.0020422 (PMC532390; doi:10.1371/journal.pbio.0020422)
Supplement: Table S1 — Also available at http://genes.mit.edu/NielsenEtAl/. (4.3 MB ZIP). [file pbio.0020422.st001.zip › NielsenEtAl/html/1384.html]

AN1383.1.NCU03860.1.MG07265.1.FG09990.1


```
 CLUSTAL W (1.82) Multiple Sequence Alignments - Introns Inserted


Sequence 1: MG07265.1	166 aa
Sequence 2: FG09990.1	152 aa
Sequence 3: NCU03860.1	167 aa
Sequence 4: AN1383.1	166 aa
Alignment Length: 178 aa
Number Identitical Residues: 56 aa
Alignment Score (without introns) 2877


MG07265.1 	MNPHQKNKIDIT0SLSPEEQRLFRLYGKLPSKSDHFAKHLK~ERKYFDSGDYALSKAGKA
NCU03860.1	MIATLTVRQISQ~SLSPEEQRLFRLYGKLPSRSDHFAKHLK~ERKYFDSGDYAMSKAGKG
FG09990.1 	MNPHQKNKVDVK0SLTPEEQRLFRLYGKLPSRSDHFAKHLK~DRKYFDSGDYAMSKAGKG
AN1383.1  	MNPHQQNKIDTS0KLSPDEQRLLRLYGKMPNKKDLLQNKLK0ERKYFDSGDYALSKAGKA
          	* .    :     .*:*:****:*****:*.:.* : ::** :**********:*****.

MG07265.1 	DSVDAGSVGSQHPVPENIPHLSSP---VNGAGSSGLIPN---------LQATGGREQQQA
NCU03860.1	DGVDTGSVGSQHPVPENIPHLTNPCGAINGASGLAQAHH---------IVHQHSNMGPQA
FG09990.1 	DGVDAGAVGSQHPLPENIPHLSSP---VNGSGTN--VP-----------KHHGSVPGIQA
AN1383.1  	SDVGVTNIGSQHPVPENIPHLTATSPGANAAGNGGSISAQGGQHIPGSISSHPGSVGFQS
          	..*..  :*****:*******: .. . *.:.  .    ....  ..:     .    *:

MG07265.1 	SRSPVKEGSFLHRETSADDDDSASNDVAENKVAAEAVGDATAAAAAAQTGGKTEEIPIRR
NCU03860.1	G-SPVKESSYFSRGLSAEEIDALNSN-EHNAEARQQQEQAAADTISAAPADQQEPIPIRQ
FG09990.1 	G-SPIKESSFLKSETSVDETQGDNVDKPQG-------ADAAAAPEPVAAGG--EGIPIRR
AN1383.1  	R-SPVKEGSFLHRGRSVDESD----NVPHT-------ADNKGSDEAVSPPPATEGVPIRR
          	  **:**.*::    *.:: :    :  .         :  .   .. .    * :***:

MG07265.1 	
FG09990.1 	
NCU03860.1	
AN1383.1  	
          	
```
